# Autopsies at Central Park Menagerie

**Published:** 1885-01

**Authors:** 


### CASE DEPARTMENT.

### I.—AUTOPSIES AT CENTRAL PARK MENAGERIE.

*Beaver* (*Castor fiber*).—Young male received from Central Park Zoölogical Gardens, Oct. 6, 1884, at 12.30 P. M.; autopsy at 3.15 P. M. same day.

Body well nourished, neglected (?), and covered by fly maggots in several places; evidently not longer dead than twenty-four hours. It was noticed, on opening the abdominal cavity, that granular matter (grains of wheat, sand, husks of barley and oats) were lying in the abdominal cavity. Further examination revealed the existence of *a large perforation* of the splenic end of the larger curve of the stomach. The perforation was shaped irregularly, covering the area of a three-cent (silver) piece; a bridge of stomach substance, bifid at its verteo mesal end, ran across it, so that in reality there were two larger subdivisions, and one smaller opening.

The edges of the opening were of a greenish gray color. This discoloration extended around the opening for nearly a centimetre.

The external (peritoneal) coat of the intestines was injected, food in large quantities was found among the intestinal folds. It showed exactly the same characters as the food contents of the stomach, and had evidently escaped from the aforesaid openings; from the latter a bolus of husks, grains, sand, and mucus was found protruding.

The liver was fatty—a normal occurrence at this season in animals destined to hibernate. The heart was perfectly healthy, likewise the kidneys.

The nerve-centres, brain, and spinal cord were pulpy, and did not show the consistency they should *caeteris paribus* have shown in so fresh a body. The pia mater was injected both in its spinal and its cerebral portions. This softness and injection of the nerve-centres and their membranes has been frequently found in animal and human subjects who have died of surgical shock, associated with septic states, or in the early stages of peritonitis.

In the subcutaneous cellular tissue of the thorax, abdomen, and neck, hemorrhages and grayish infiltrated spots were observed, such as are characteristic of a scorbutic state. There was an ulcer in one of the cœcal appendages.

*Anatomico-Pathological Diagnosis.*—Scorbutus; necrotic or gangrenous ulceration of the stomach; perforation of the gastric wall; escape of gastric contents into the abdominal cavity, and death by shock from the inflammatory involvement of the peritoneum.

*Dog-faced Baboon* (*Cynocephalus porcarius*).—Middle-aged, fair condition, tubercular infiltration of the peribronchial glands; several spots of "reactive" inflammation in the lungs; intestines contracted and atrophied. There was an exudation of blood, mingled with serum, under the pia mater and in the upper layers of the cortical brain substance, extending into the sulci. This was strictly limited to an area of the size of half a dollar, covering the first, second, third, and fourth gyri of the temporo-sphenoidal lobe of the left side, near the apex of the latter. The consistency of the tissues of the brain and spinal cord generally was softer than usual, but there was no disease of the blood vessels to account for the hemorrhage. It suggested the possible causation by a blow or fall. ***

